# Vaspin inhibits cytokine-induced nuclear factor-kappa B activation and adhesion molecule expression via AMP-activated protein kinase activation in vascular endothelial cells

**DOI:** 10.1186/1475-2840-13-41

**Published:** 2014-02-12

**Authors:** Chang Hee Jung, Min Jung Lee, Yu Mi Kang, Yoo La Lee, Hae Kyeong Yoon, Sang-Wook Kang, Woo Je Lee, Joong-Yeol Park

**Affiliations:** 1 Department of Internal Medicine, University of Ulsan College of Medicine, Poongnap-dong, Songpa-gu, Seoul 138-736, Korea; 2 Asan Institute of Life Sciences, University of Ulsan College of Medicine, Seoul, Korea; 3 Department of Biomedical Sciences, University of Ulsan College of Medicine, Seoul, Korea

**Keywords:** Vaspin, Endothelial cells, AMPK, NF-κB, Adhesion molecules

## Abstract

**Background:**

Vaspin is an adipocytokine that was recently identified in the visceral adipose tissue of diabetic rats and has anti-diabetic and anti-atherogenic effects. We hypothesized that vaspin prevents inflammatory cytokine-induced nuclear factor-kappa B (NF-κB) activation by activating AMP-activated protein kinase (AMPK) in vascular endothelial cells.

**Methods:**

We examined the effects of vaspin on NF-κB activation and the expression of the NF-κB-mediated genes intercellular adhesion molecule-1 (ICAM-1), vascular cell adhesion molecule-1 (VCAM-1), E-selectin, and monocyte chemoattractant protein-1 (MCP-1). Human aortic endothelial cells (HAECS) were used. Tumor necrosis factor alpha (TNFα) was used as a representative proinflammatory cytokine.

**Results:**

Treatment with vaspin significantly increased the phosphorylation of AMPK and acetyl-CoA carboxylase, the down-stream target of AMPK. Furthermore, treatment with vaspin significantly decreased TNFα-induced activation of NF-κB, as well as the expression of the adhesion molecules ICAM-1, VCAM-1, E-selectin, and MCP-1. These effects were abolished following transfection of AMPKα1-specific small interfering RNA. In an adhesion assay using THP-1 cells, vaspin reduced TNFα-induced adhesion of monocytes to HAECS in an AMPK-dependent manner.

**Conclusions:**

Vaspin might attenuate the cytokine-induced expression of adhesion molecule genes by inhibiting NF-κB following AMPK activation.

## Introduction

Vascular inflammation is a primary event in the pathogenesis of many human diseases, including atherosclerosis, hypertension, and restenosis [1-3]. The vascular inflammatory reaction is mediated by complex interactions between circulating leukocytes and the endothelium. Activation of endothelial cells by proinflammatory molecules, including tumor necrosis factor α (TNFα), increases the expression of adhesion molecules and the adhesion of leukocytes to the vascular endothelium, which are critical steps in the initiation of atherosclerosis [4]. Vascular adhesion molecules are critical for the initiation and progression of atherosclerosis [5].

The transcription factor nuclear factor-κB (NF-κB) is involved in a wide variety of phenomena, including atheroscleorosis [6]. Activated NF-κB has been identified in situ in human atherosclerotic plaques [7,8]. A number of genes of which the products have been implicated in the development of atherosclerosis are regulated by NF-κB. Various leukocyte adhesion molecules, such as intracellular adhesion molecule-1 (ICAM-1), vascular adhesion molecule-1 (VCAM-1), and E-selectin, as well as various chemokines such as monocyte chemoattractant protein-1 (MCP-1) and IL-8 have been reported to promote atherosclerosis through the NF-κB-dependent coordinated induction [9-12].

The enzyme AMP-activated kinase (AMPK) is activated when cellular energy is depleted [13]. Although the AMPK pathway has been traditionally regarded as an intracellular fuel gauge and a regulator of metabolism, recent evidence suggests that it also protects endothelial function [14]. AMPK has pleiotrophic effects that may be anti-atherogenic and beneficial to endothelial function, including an anti-inflammatory effect through its suppression of cytokine-induced NF-κB activation in vascular endothelial cells [14,15].

Adipose tissue is not only a tissue in which energy is stored, but is also an active endocrine organ that can release a variety of cytokines termed adipocytokines [16]. Vaspin (visceral adipose tissue-derived serine protease inhibitor), a member of the serine protease inhibitor family, is a novel 392–395-amino acid adipocytokine identified in visceral white adipose tissues of the Otsuka Long-Evans Tokushima Fatty rat, an animal model of abdominal obesity with type 2 diabetes [17]. We have recently reported that vaspin has anti-atherogenic properties such as its anti-apoptotic effect against free fatty acid in vascular endothelial cells and its positive effect on nitric oxide (NO) bioavailability [18,19]. Besides these anti-atherogenic effects of vaspin, recombinant human vaspin increases the phosphorylation of AMPK in hepatocytes, thereby exerting a protective effect against diet-induced obesity, glucose intolerance, and hepatic steatosis [20].

In the present study, we hypothesized that vaspin prevents NF-κB activation in vascular endothelial cells that are exposed to inflammatory cytokines. We examined the effects of vaspin on NF-κB activation, as well as on the expression of the NF-κB-mediated genes ICAM-1, VCAM-1, E-selectin, and MCP-1 in vascular endothelial cells. We also examined whether AMPK activation mediates the effect of vaspin on NF-κB activation and the subsequent alterations in expression of NF-κB-mediated genes.

## Materials and methods

### Cell culture and treatment

Human aortic endothelial cells (HAECs) were obtained from Lonza Inc. (CC-2535, Walkersville, MD, USA) and maintained in endothelial basal medium (CC-3162, Lonza) supplemented with various growth factors that are required for the growth of endothelial cells and 2% fetal bovine serum at 37°C in a humidified incubator supplemented with 5% CO_2_.

THP-1 cells, a human monocytic cell line (ATCC® TIB-202™, Rockville, MD, USA), were grown in RPMI-1640 medium containing 10% fetal bovine serum. In all experiments, cells were used at six or fewer passages.

TNFα (210-TA-020, R&D Systems, Inc. Minneapolis, MN, USA) was used as the representative proinflammatory cytokine and phosphate buffered saline was used as the vehicle. Cells were transferred to medium containing 1% fetal bovine serum and incubated in media containing 10 ng/mL TNFα for the indicated amount of time. Vaspin was obtained from Adipogen Inc. (AG-40A0064, Incheon, Korea) and phosphate buffered saline was used as the vehicle. Cells were transferred to medium containing 1% fetal bovine serum and incubated in media containing various concentrations of vaspin for the indicated amount of time before treatment with TNFα. 5-Aminoimidazole-4-carboxamide-1-β-d-ribofuranoside (123040, Calbiochem, Darmstadt, Germany), an activator of AMPK, was used as a positive control.

### Western blot analysis

HAECs treated with TNFα in the presence or absence of vaspin for the indicated amount of time were lysed in cell lysis buffer (#9803, Cell Signaling, Danvers, MA, USA) containing 1 mM PMSF. After lysing cells, protein samples (20 μg/lane) were resolved by electrophoresis on 10% sodium dodecyl sulfate-polyacrylamide gels and then transferred to nitrocellulose membranes. Membranes were incubated in blocking buffer and then with one or more of the following primary antibodies: anti-AMPK (#2532, Cell Signaling, 1:1000), anti-phosphorylated AMPK (#2531, Thr172, Cell Signaling, 1:1000), anti-acetyl-CoA carboxylase (#3662, ACC, Cell Signaling, 1:1000), anti-phosphorylated ACC (#3661, Ser79, Cell Signaling, 1:1000), anti-phosphorylated Akt (#9271, Cell Signaling, 1:1000), anti-ICAM-1 (#4915, Cell Signaling, 1:1000), anti-VCAM-1 (#12367, Cell Signaling, 1:1000), anti-E-selectin (NBP1-45545, Novus Biologicals, CO, USA), and anti-MCP-1 (ab25124, Abcam, Cambridge, UK, 1:1000) antibodies.

For the inhibitor of NF-κB (IκB) experiments, membranes were incubated with anti-IκBα (#4812, Cell Signaling, 1:1000), anti-phosphorylated IκBα (#4812, Ser32, Cell Signaling, 1:1000), or anti-β-actin (A5316, Sigma, St. Louis, MO, USA, 1:10,000) antibodies. After incubating with primary antibodies, membranes were washed and incubated with horseradish peroxidase-conjugated secondary antibodies (PI1000, Vector laboratories Inc. Burlingame, CA, USA). Immunoreactive bands were visualized by enhanced chemiluminescence (RPN2106, Amersham Bioscience, UK).

### NF-κB activation

To measure NF-κB activation, HAECs were transfected with a cis-reporter plasmid containing the luciferase reporter gene linked to five repeats of NF-κB binding sites (pNF-κB Luc: 219077, Stratagene, LaJolla, CA, USA) using LipofectAMINE 2000 (11668019, Invitrogen, Carlsbad, CA, USA), as previously described [21]. pRL-SV40 renilla luciferase control reporter vector (10 ng, E2231, Promega, Madison, WI, USA) was co-transfected as an internal control. Twenty-four hours after transfection, cells were unstimulated or stimulated with 100 ng/mL vaspin and harvested 4 hrs later. Luciferase activity was measured using a Dual-Luciferase Reporter Assay (E1910, Promega) and normalized to renilla luciferase activity.

Changes in the levels of p50 and p65, two crucial subunits of activated NF-κB [22], were also measured in nuclear extracts from HAECs using a Transcription Factor Assay Kit (41096, 40096, Active Motif Japan, Tokyo, Japan). Nuclear extracts were prepared using a Nuclear/Cytosol Fractionation Kit (#K266-100, BioVision, Milpitas, CA, USA), after which levels of p50 and p65 were quantified using recombinant p50 and p65 proteins (Active Motif) as the standards.

### Transfection of small interfering RNA (siRNA)

An AMPKα1-specific siRNA (sc-29673, Santa Cruz Biotechnology, Inc., Santa Cruz, CA, USA) or a control siRNA (sc-37007, Santa Cruz Biotechnology) were used to examine whether AMPK mediated the inhibitory effect of vaspin on TNFα-induced changes in NF-κB activity as well as the subsequent increased expression of adhesion molecules in HAECs. HAECs were transfected with 10 nM anti-AMPKα1 siRNA or control siRNA using LipofectAMINE 2000 (Invitrogen) 48 hrs before vaspin treatment.

### Real-time PCR analysis

cDNAs were synthesized using the ReverTra ACE qPCR RT kit (FSQ-101, Toyobo, Osaka, Japan). Real-time analysis was performed on an ABI 7500 Fast RT-PCR system (Foster City, CA, USA) with the Fast SYBR® Green Master Mix (4385612, Applied Biosystems, CA, USA). Each sample was assayed in duplicate in a 20 μL reaction volume containing 1 μL cDNA (corresponding to 100 ng of total RNA input), 10 μL 2X SYBR Green master mix (Applied Biosystems), and 1 μL of forward and reverse primers (10 pmol/μL for each). Negative controls (no template or RNA) were included to ensure the absence of contamination. Amplification of 18S rRNA was used as the internal control. The ratio between the expression levels of the target gene and 18S rRNA was calculated using a relative quantification method (ΔΔ cycle value (Ct) method) as described previously (User Bulletin No. 2, Applied Biosystems).

In brief, the amplification plot is a plot of fluorescence versus PCR cycle number. The threshold Ct is the fractional PCR cycle number at which the fluorescent signal reached the detection threshold. Therefore, the input cDNA copy number and Ct are inversely related. Data were analyzed using the Sequence Detector System software version 2.1 (ABI) and the Ct value was automatically converted to the fold change RQ value. The fold change (RQ) = 2 ^− (ΔΔCT)^, where − (ΔΔCT) = − (ΔCT_trt_ − ΔCT_control_) = − [(Ct_Target_ – Ct18s)_trt_ − (Ct_Target_ – Ct18s)_control_]. The following primers were used: ICAM-1 forward 5’-ACT GCA GGC CTC AGC ACG TA-3’, reverse 5’-CGT GGC TTG TGT GTT CGG TT-3’; VCAM-1 forward 5’-CGG ATT GCT GCT CAG ATT GG-3’, reverse 5’-ACT CCT CAC CTT CCC GCT CA-3’; E-selectin forward 5’-AGA GTG GAG CCT GGT CTT ACA-3’, reverse 5’-CCT TTG CTG ACA ATA AGC ACT GG-3’; MCP-1 forward 5’-TTC CAT GGA CCA CCT GGA CA-3’, reverse 5’-TGT CTG GGG AAA GCT AGG GG-3’, and 18S rRNA forward 5’-CGC CGC TAG AGG TGA AAT TC-3’, reverse 5’-TTG GCA AAT GCT TTC GCT C-3’.

### Monocyte adhesion assay

THP-1 cells were labeled with VibrantDiO® Cell-Labeling Solution (V-22886, Molecular Probes, Grand Island, NY, USA), placed on a confluent HAEC monolayer (1 × 10^5^ HAECs per well) in a 96-well plate (1 × 10^6^ THP-1 cells per well), and allowed to adhere for 10 min. After non-adherent cells were removed, the fluorescent intensity of adhered and total cells applied to the well was measured using a fluorescence plate reader (VICTOR X2, Perkinelmer, Waltham, MA, USA). The adherent cells were visualized using confocal microscopy (LSM710, ZEISS, Germany). The ratio of adherent to total cells was expressed as ‘adhesion (%)’.

### Statistical analysis

All data are shown as mean values ± standard error of mean (SEM). Two groups were compared using an independent Student’s t-test. Multiple groups were compared using a one-way ANOVA followed by Tukey’s multiple comparison post-hoc test. A *p-*value less than 0.05 was considered statistically significant. All experiments were performed at least three times. All statistical analyses were performed using SPSS18.0 for Windows (SPSS, Inc., Chicago, IL, USA).

## Results

### Vaspin activates AMPK in HAECs

**Figure 1:**
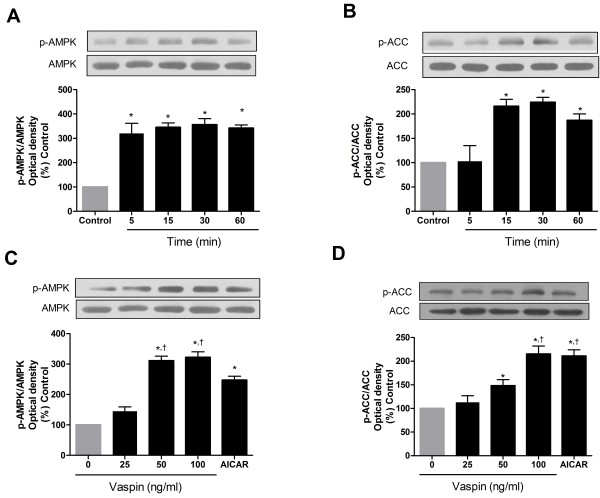
**Vaspin activates AMPK in HAECs.** HAECS were incubated with 100 ng/mL vaspin for different amounts of times **(A and B)**, or with different concentrations of vaspin or vehicle for 1 hr at 37°C **(C and D)**. Phosphorylated AMPK and ACC were detected by Western blotting using phospho-specific antibodies for AMPK and ACC (*top*). Levels of phosphorylated AMPK and ACC were normalized against total levels of AMPK and ACC (*bottom*). Data are shown as mean values ± SEM of five independent experiments. 5-Aminoimidazole-4-carboxamide-1-β-d-ribofuranoside (AICAR) was used as a positive control in C and D. ^*^p < 0.05 vs. untreated cells (Control), ^†^*p* < 0.05 vs. cells treated with 25 ng/mL vaspin.

Incubation of HAECs with vaspin (100 ng/mL) induced the activation of AMPK, as measured by the phosphorylation of AMPK (Figure 1A) and its down-stream effector, ACC (Figure 1B). Dose-response studies demonstrated that levels of phosphorylated AMPK and ACC were significantly higher in cells treated with a concentration of vaspin of 50 ng/mL or higher than in control cells (Figure 1C and D).

### Vaspin inhibits TNFα-induced NF-κB activation

**Figure 2:**
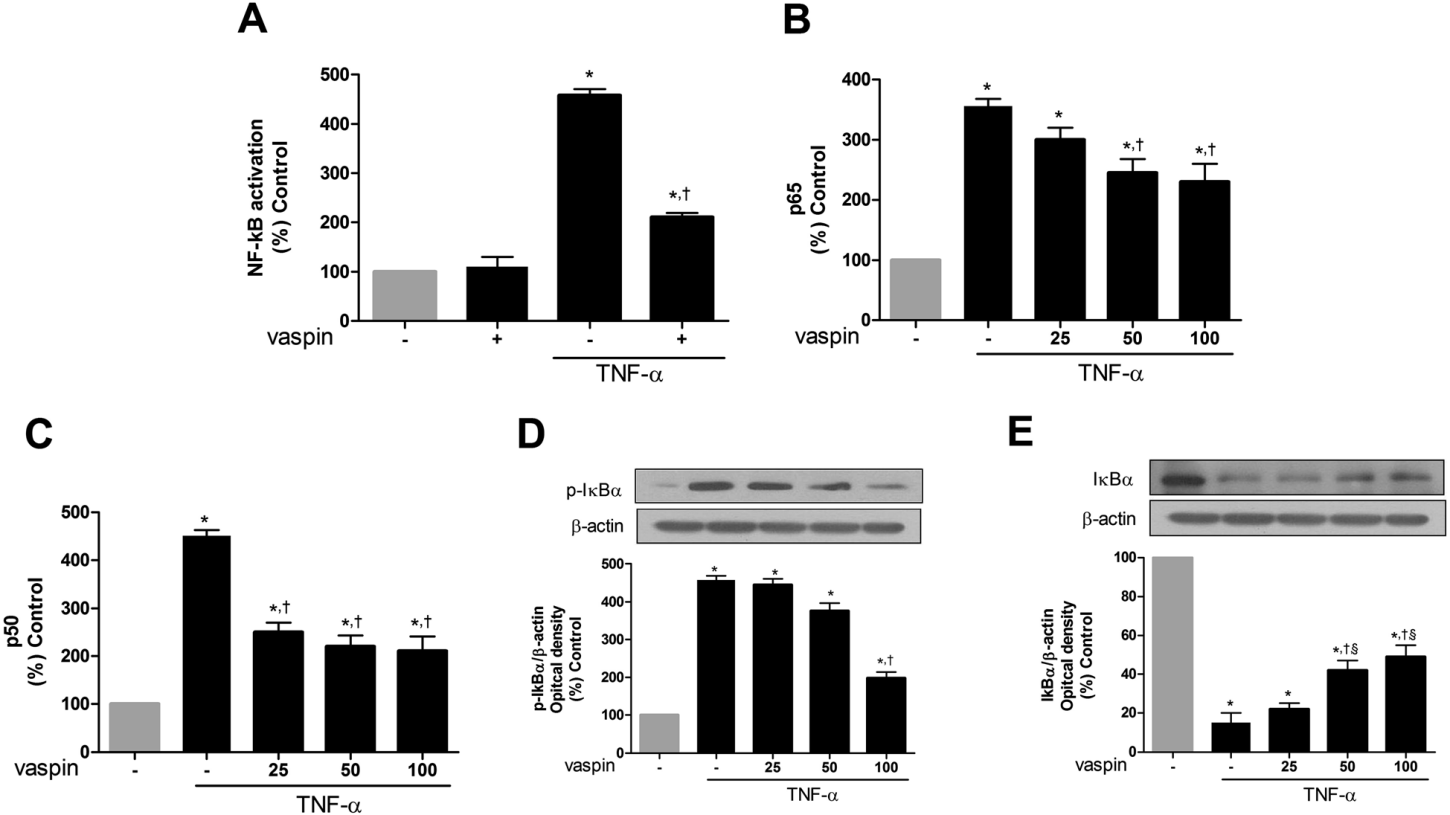
**Vaspin inhibits TNFα-induced NF-κB activation in HAECs.** The effect of vaspin on TNFα-induced NF-κB promoter activity **(A)**. NF-κB promoter activity was measured at 4 hr after treatment with 100 ng/mL vaspin in the presence or absence of 10 ng/mL TNFα. The effects of vaspin on TNFα-induced activation of p65 **(B)**, and p50 **(C)**. Standardized amounts of p65 **(B)** and p50 **(C)** were measured at 1 hr after treatment with 25–100 ng/mL vaspin in the presence or absence of 10 ng/mL TNFα. The effect of vaspin on TNFα-induced IκBα degradation **(D and E)**. Relative levels of phosphorylated IκBα **(D)** and phosphorylated IκBα **(E)** were measured at 30 min after treatment with 25–100 ng/mL vaspin in the presence or absence of 10 ng/mL TNFα. Data are mean values ± SEM of five independent experiments. ^*^p < 0.05 vs. untreated cells, ^†^*p* < 0.05 vs. cells treated with TNFα alone, and ^§^*p* < 0.05 vs. cells treated with 25 ng/mL vaspin.

We next examined the effect of vaspin on TNFα-induced NF-κB activation in HAECs. Treatment with TNFα induced an approximately 4.5-fold increase in NF-κB-mediated reporter gene expression (Figure 2A). However, pretreatment with vaspin (100 ng/mL) significantly inhibited this TNFα-induced NF-κB activation (Figure 2A).

We also measured levels of p50 and p65 in nuclear extracts from untreated HAECs and from HAECs cultured with TNFα in the presence (25–100 ng/mL) or absence of vaspin. Levels of p50 and p65 were markedly increased after treatment with TNFα (Figure 2B and C); however, these increases were significantly inhibited by vaspin treatment (Figure 2B and C).

To confirm the effect of vaspin on TNFα-induced NF-κB activation, we measured the protein levels of IκBα and phosphorylated IκBα. As previously reported [23], treatment with TNFα induced phosphorylation of IκBα in HAECs (Figure 2D). The blot was then re-probed with an anti-IκBα antibody, which indicated that IκBα was markedly degraded following TNFα treatment (Figure 2E). Vaspin treatment partially inhibited this TNFα-induced degradation of IκBα (Figure 2D and E). Collectively, these results demonstrated that treatment with vaspin significantly inhibited TNFα-induced NF-κB activation in HAECs.

### Vaspin inhibits TNFα-induced expression of adhesion molecules

**Figure 3:**
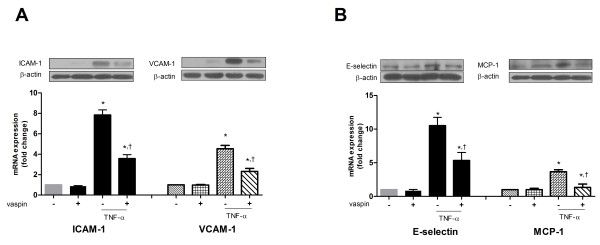
**Vaspin inhibits TNFα-induced expression of adhesion molecules.** The effect of vaspin on TNFα-induced expression of ICAM-1 **(A)**, VCAM-1 **(A)**, E-selectin **(B)**, and MCP-1 **(B)**. The protein level and mRNA expression of each adhesion molecule was measured at 24 hr after treatment with 100 ng/mL vaspin in the presence or absence of 10 ng/mL TNFα. Data shown are representative Western blots (top panels) and mean values ± SEM of three independent experiments (bottom panels). The fold change in mRNA expression compared to levels in untreated cells is shown. ^*^p < 0.05 vs. untreated cells, and ^†^*p* < 0.05 vs. cells treated with TNFα alone.

Based on the inhibition of TNFα-induced NF-κB activation by vaspin treatment, we next examined the effects of vaspin on TNFα-induced expression of ICAM-1, VCAM-1, E-selectin, and MCP-1 at both levels of protein and mRNA. Incubation of cells with TNFα for 24 hrs significantly increased the expression of ICAM-1, VCAM-1 (Figure 3A), E-selectin, and MCP-1 (Figure 3B) at both levels of protein and mRNA. However, pretreatment with vaspin (100 ng/mL) significantly inhibited the TNFα-induced expression of ICAM-1, VCAM-1 (Figure 3A), E-selectin, and MCP-1 (Figure 3B).

**Figure 4:**
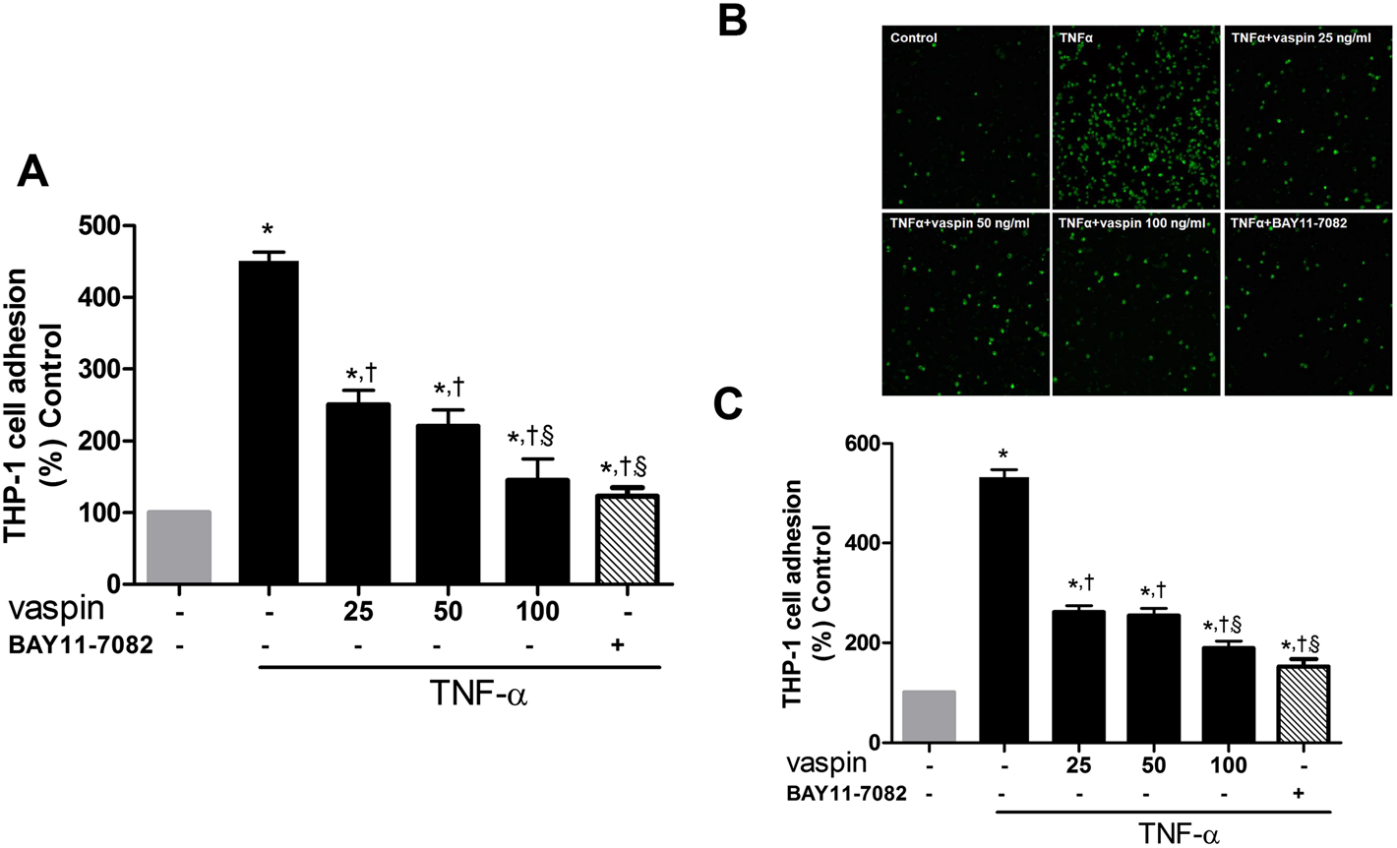
**Vaspin inhibits TNFα-induced adhesion of monocytes to HAECs.** The effect of vaspin on TNFα-induced THP-1 cell adhesion to HAECS measured using a static adhesion assay **(A)** and staining with VibrantDiO® Cell-Labeling Solution **(B and C)**. The level of THP-1 cell adhesion to HAECs was measured at 4 hr after treatment with 25–100 ng/mL vaspin in the presence or absence of 10 ng/mL TNFα. BAY11-7082 (10 μM, S2913, Selleckchem, USA), an inhibitor of NF-κB, was used as a positive control. In **A** and **C**, data shown are mean values ± SEM of three independent experiments. Representative microphotographs are shown in B, and these data are quantified in C. Green fluorescence was visualized using confocal microscopy. Magnification, × 40. ^*^p < 0.05 vs. untreated cells, ^†^*p* < 0.05 vs. cells treated with TNFα alone, and ^§^*p* < 0.05 vs. cells treated with 25 ng/mL vaspin.

Pretreatment of HAECs with vaspin (25–100 ng/mL) for 4 hrs significantly inhibited the TNFα-induced adhesion of THP-1 cells to HAECs in a dose-dependent manner (Figure 4). This inhibitory effect of vaspin on TNFα-induced monocyte adhesion to HAECs was comparable to that of BAY11-7082, an NF-κB inhibitor (Figure 4).

### AMPK activation plays a role in the inhibitory effects of vaspin on TNFα-induced NF-κB activation and expression of adhesion molecules

**Figure 5:**
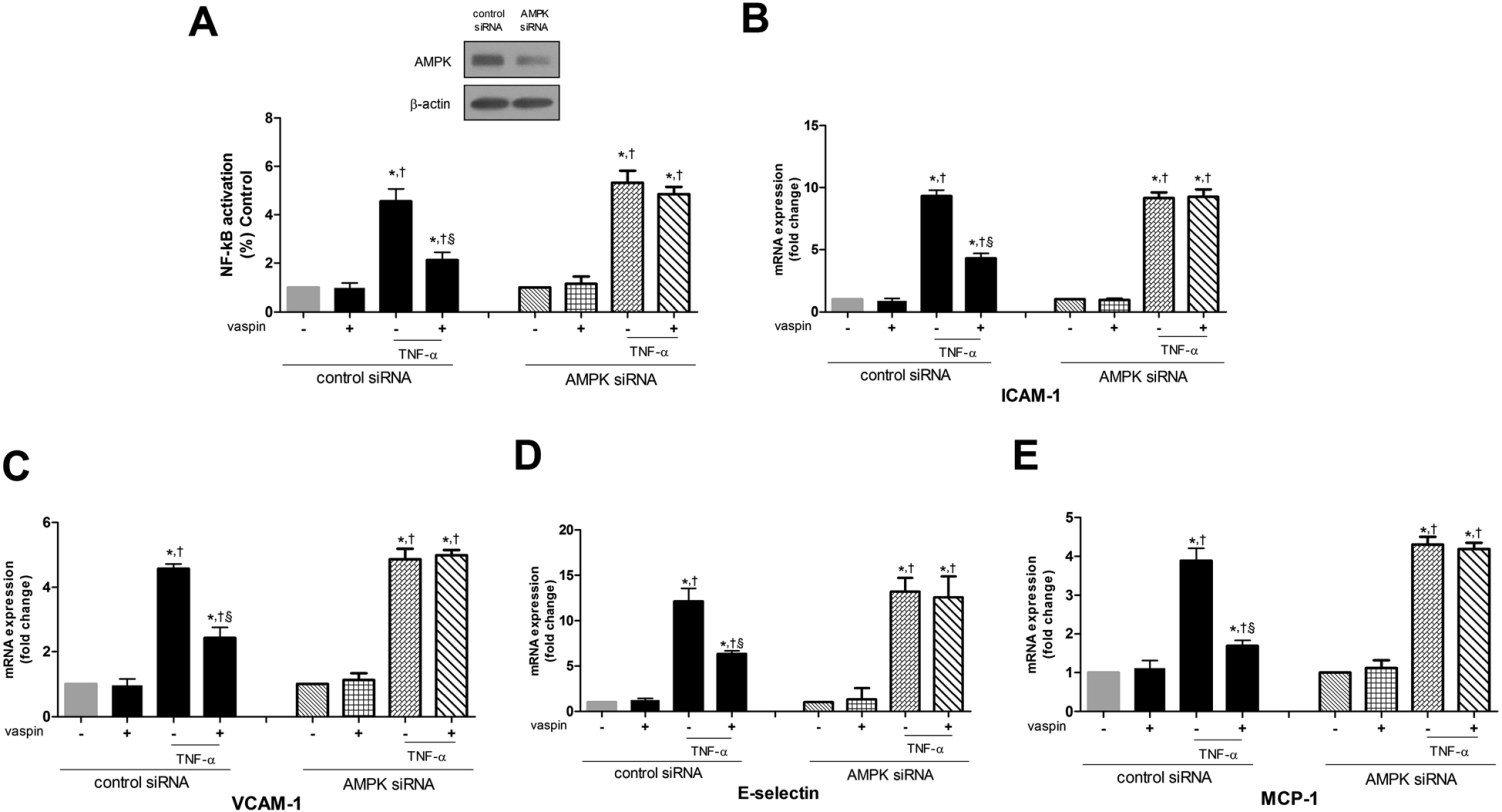
**AMPK activation mediates the inhibitory effects of vaspin on TNFα-induced NF-κB activation and expression of adhesion molecules.** The effects of vaspin on TNFα-induced NF-κB promoter activity **(A)** and mRNA expression of the adhesion molecules ICAM-1 **(B)**, VCAM-1 **(C)**, E-selectin **(D)**, and MCP-1 **(E)** were measured at 24 hr after treatment of HAECs with 100 ng/mL vaspin in the presence of absence of 10 ng/mL TNFα. HAECs were transfected with control siRNA or AMPKα1 siRNA 24 hr before. Data shown are mean values ± SEM of five independent experiments. ^*^p < 0.05 vs. untreated cells, ^†^*p* < 0.05 vs. cells treated with vaspin alone, and ^§^*p* < 0.05 vs. cells treated with TNFα alone.

Finally, we examined the role of AMPK activation in the inhibitory effects of vaspin on cytokine-induced NF-κB activation and the subsequent increased gene expression of adhesion molecules. The inhibitory effect of vaspin on TNFα-induced NF-κB activation in control siRNA-treated cells was abolished in AMPKα1 siRNA-transfected cells (Figure 5A). Similarily, the inhibitory effect of vaspin on TNFα-induced expression of the adhesion molecules ICAM-1 (Figure 5B), VCAM-1 (Figure 5C), E-selectin (Figure 5D), and MCP-1 (Figure 5E) was almost completely abolished in cells transfected with AMPKα1 siRNA. These results indicate that AMPK activation mediates the inhibitory effects of vaspin on TNFα-induced NF-κB activation and expression of adhesion molecules.

**Figure 6:**
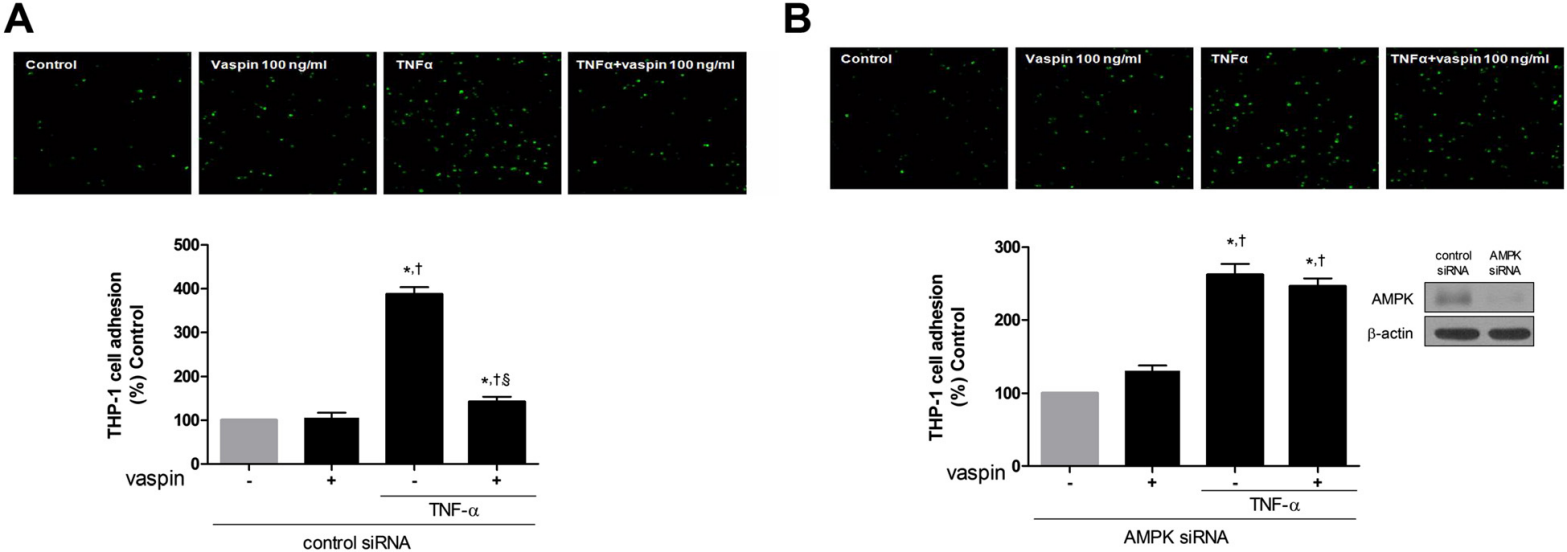
**AMPK activation mediates the inhibitory effects of vaspin on TNFα-induced adhesion of monocytes to HAECs.** The effect of vaspin on TNFα-induced adhesion of monocytes to HAECs was measured by staining with VibrantDiO® Cell-Labeling Solution at 4 hr after treatment with 100 ng/mL vaspin in the presence of absence of 10 ng/mL TNFα. HAECs were transfected with control siRNA **(A)** or AMPKα1 siRNA **(B)** 24 hr before. Data shown are representative microphotographs (top panels) and mean values ± SEM of three independent experiments (bottom panels). Green fluorescence was visualized using confocal microscopy. Magnification, × 40. ^*^p < 0.05 vs. untreated cells, ^†^*p* < 0.05 vs. cells treated with vaspin alone, and ^§^*p* < 0.05 vs. cells treated with TNFα alone.

Similarily, the inhibitory effect of vaspin on TNFα-induced monocyte adhesion to HAECs was abolished in cells transfected with AMPKα1 siRNA (Figure 6). This supports the role of AMPK in the inhibitory effect of vaspin on TNFα-induced expression of adhesion molecules.

### Vaspin activates AMPK independently of Akt and NO pathway

**Figure 7:**
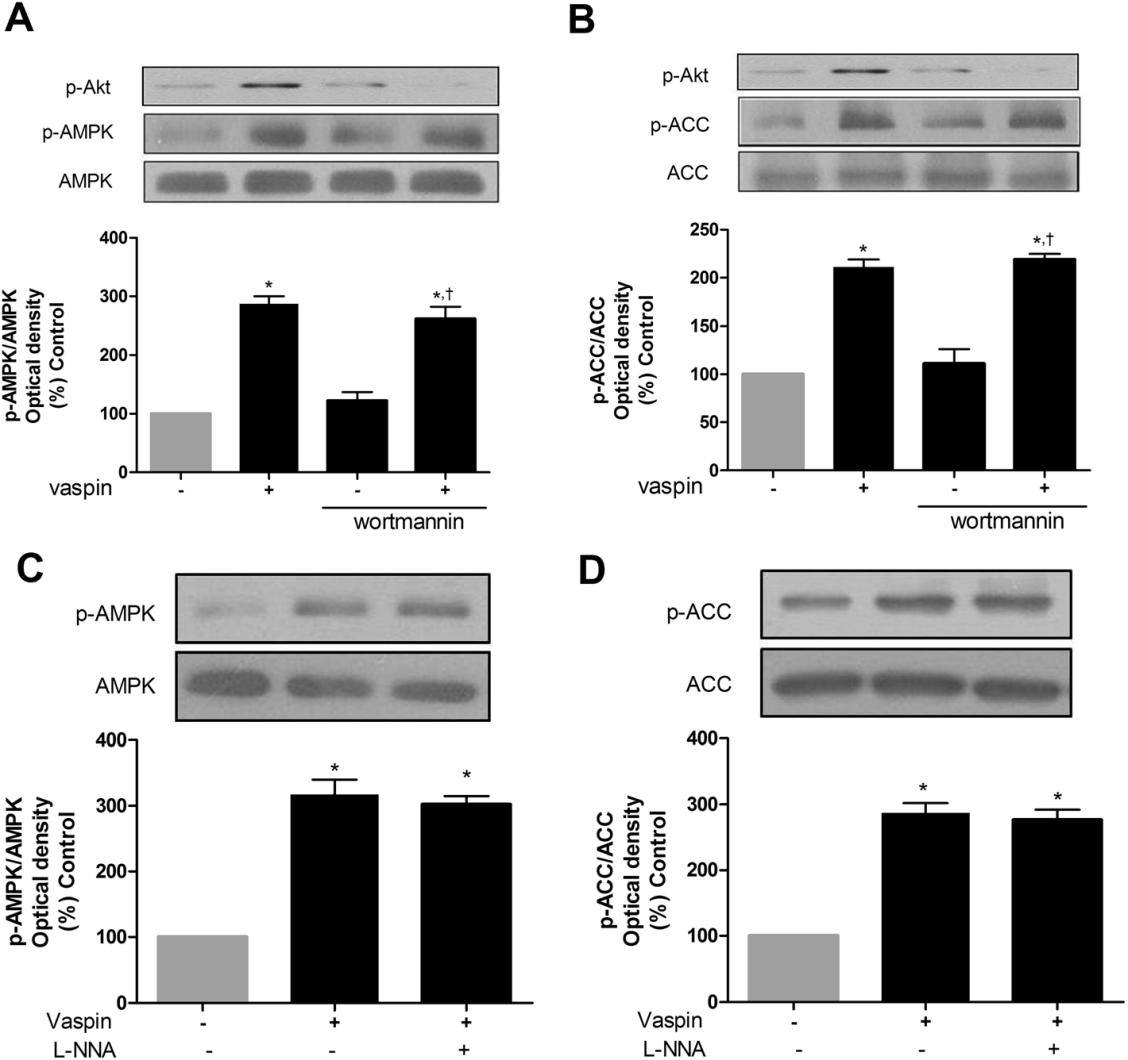
**Vaspin induces the activation of AMPK independently of Akt and NO pathway.** HAECS were incubated with 100 ng/ml vaspin or vehicle for 1 hr at 37°C. Wortmannin (100 nM, 681675, Calbiochem, Darmstadt, Germany), a specific covalent inhibitor of PI3-kinase or nitro-L-arginine (500 μM, L-NNA, N5501, Sigma), an eNOS inhibitor, was pretreated 15 min before the vaspin treatment. Phosphorylation of AMPK **(A and C)**, and ACC **(B** and **D)** were detected by Western blotting using phospho-specific antibodies for AMPK and ACC, normalized to total AMPK and ACC. Data are shown as means ± SEM of three independent experiments. AMPK, AMP-activated protein kinase; NO, nitric oxide; ACC, acetyl-CoA carboxylase; N-NNA, nitro-L-arginine; eNOS, endothelial nitrix oxide synthase. In **A** and **B**, ^*^p < 0.05 vs. untreated (Control), ^†^*p* < 0.05 vs. cells treated with wortmannin alone. In **C** and **D**, ^*^p < 0.05 vs. untreated (Control).

Previously, it has been demonstrated that vaspin activates Akt via its stimulatory effect on phosphatidylinositol3-kinase (PI3-kinase), which may be linked to activation of endothelial nitric oxide synthase (eNOS), increased production of NO and activation of AMPK [19,20,24]. Furthermore, we have recently demonstrated that vaspin increased NO bioavailability through its suppressive effect on the production of asymmetric dimethylarginine, an endogenous eNOS inhibitor [18]. To examine the possible role of these molecules (i.e., Akt and eNOS), we measured the phosphorylation of AMPK and ACC after pretreatment cells with wortmannin, a specific covalent inhibitor of PI3-kinase or nitro-L-arginine, an eNOS inhibitor [25]. As shown in Figure 7, the stimulatory effect of vaspin on the AMPK activation was independent of its stimulatory effect on PI3-kinase/Akt pathway (Figure 7A and B) and increased NO bioavailability (Figure 7C and D).

## Discussion

In the present study, we demonstrated that vaspin inhibits TNFα-induced NF-κB activation in vascular endothelial cells. Vaspin thereby inhibited NF-κB-dependent gene expression of the inflammatory and cell adhesion molecules ICAM-1, VCAM-1, E-selectin, and MCP-1. Furthermore, we investigated whether activation of AMPK by vaspin contributes to this inhibition of cytokine-induced NF-κB activation and expression of cell adhesion molecules. Transfection of AMPK α1 siRNA, which reduced AMPK expression by about 80% (Figure 5A), significantly attenuated vaspin-induced inhibition of TNFα-induced NF-κB activation and gene expression of various adhesion molecules in vascular endothelial cells. These data suggest that AMPK activation is responsible for vaspin-induced inhibition of NF-κB activation. Furthermore, this stimulatory effect of vaspin on the AMPK activation was independent of Akt and NO pathway, two representative signaling pathways linked to vaspin in vascular endothelial cells [18,19,26].

Although we did not perform a kinase assay of AMPK, vaspin likely activates AMPK. This is because the extent of AMPK phosphorylation at Thr172 strongly reflects its activity [27], and the consensus AMPK down-stream effector ACC [28] was phosphorylated at Ser79 in vaspin-treated cells (Figure 1B and D). How vaspin activates AMPK in vascular endothelial cells remains to be elucidated; however, our data are in agreement with the effect of vaspin on AMPK activation in hepatocytes [20].

Vaspin was recently reported to prevent TNFα-induced ICAM-1 expression through its inhibitory effect on reactive oxygen species-dependent NF-κB activation in vascular smooth muscle cells [29]. Furthermore, vaspin attenuates the induction of cytokines and vascular smooth muscle cell proliferation by high glucose levels [30]. Vaspin elicits these effects by preventing the generation of reactive oxygen species and inhibiting NF-κB signaling [30]. AMPK has pleiotrophic effects that may be anti-atherogenic, including the suppression of reactive oxygen species production induced by deleterious stimuli, such as hyperglycemia or high levels of free fatty acids [14,31]. AMPK also has an anti-inflammatory effect through its inhibitory effects on free fatty acids or cytokine-induced NF-κB activation [14,32]. Considering these effects, AMPK might be the upstream signaling molecule through which vaspin exerts its anti-atherogenic effect in vascular smooth muscle cells [29,30]. Although further studies are required to investigate this hypothesis, our study is meaningful in that we firstly demonstrated the possible role of AMPK activation by vaspin in the aforementioned vaspin’s anti-inflammatory effect in vascular endothelial cells.

NF-κB is rapidly activated by various agents including inflammatory cytokines, such as TNFα, and is involved in a wide variety of biological phenomena including inflammatory and immune responses [22]. Upon stimulation with various stimuli, the IκB kinase complex is activated and phosphorylates specific serine residues within IκB, which triggers the degradation of IκB and the subsequent nuclear translocation of p65 and p50 [22]. We demonstrated that vaspin inhibits the gene expression of proinflammatory and adhesion molecule genes by blocking TNFα-induced phosphorylation and subsequent degradation of IκBα (Figure 2D and E). These data suggest that vaspin might suppress TNFα-induced NF-κB activation before IκB phosphorylation. Although we did not examine the effect of vaspin on IκB kinase activity, the decreased activity of IκB kinase through vaspin-mediated AMPK activation might be the possible mechanism, considering the previous results which showed that AMPK can directly phosphorylate and attenuate the IκB kinase activity [24,33,34].

Several therapeutic approaches have been tried to ameliorate the endothelial injury by modulating the NF-κB-mediated signaling pathways such as knocking-down ultimate downstream effector and/or through the development of chemicals which help to reduce the NF-κB activity triggered by harmful stimuli. For example, knocking down the profilin-1, an actin-binding protein as well as the ultimate downstream effector in endothelial injury mediated by advanced glycation end products via NF-κB, attenuated the extent of endothelial abnormalities by reducing ICAM-1 and elevating NO levels [35]. Regarding chemicals, propofol, a widely used intravenous anesthetic agent, has been demonstrated to decrease NF-κB activity, attenuated high glucose-induced endothelial adhesion molecules expression such as ICAM-1, VCAM-1 and E-selectin and mononuclear-endothelial adhesion [36]. Vaspin can be considered as one of those molecules possessing anti-inflammatory effect via down-regulating the NF-κB pathway.

Recently, a cell-surface GRP78/voltage-dependent anion channel complex is considered as a potential receptor for vaspin in endothelial cells [26]. Although the role of this GRP78/voltage-dependent anion channel complex in the activation of AMPK in vascular endothelial cells remains unclear, we could identify the role of GRP78 in the activation of AMPK pathway by vaspin in vascular endothelial cells by knocking down the expression of GRP78 using GRP78-specific siRNA (Online Additional file 1: Figure S1). Further research is needed to elucidate how vaspin interacts with GRP78 and leads to the activation of AMPK in vascular endothelial cells.

AMPK is a heterotrimeric protein consisting of three subunits, α, β, and γ, each of which has at least two isoforms, which means that 12 complexes can form [14]. These combinations generate AMPK complexes with different properties and tissue specificities [37]. The α-subunit contains the catalytic site, whereas the β- and γ-subunits are important for maintaining the stability of the heterotrimer complex [38]. The α1 isoform of AMPK is the predominant form in endothelial cells, although the α1 and α2 catalytic subunits are also expressed in these cells [39,40]. This is why we knocked down the α1 isoform of AMPK using siRNA.

In the carotid arteries of a balloon injury rat model, treatment with an adenovirus vector expressing vaspin significantly suppresses the expression of MCP-1 and protects against vascular injuries [26], which is in agreement with the results of the current study. However, our study is the first to demonstrate the role of AMPK activation in the inhibitory effects of vaspin on cytokine-induced expression of proinflammatory molecules, including MCP-1, in vascular endothelial cells.

Emerging studies have revealed that visceral white adipocytes can act as an active endocrine tissue, secreting adipocytokines that play a key role in the relationship between obesity and the development of cardiovascular disease [41]. There are suggested to be two types of adipocytokines, namely, ‘good adipocytokines’, of which adiponectin is probably the only well-established example, and ‘bad adipocytokines’, which may include inflammatory cytokines, such as TNFα, IL-6, MCP-1, and plasminogen activator inhibitor-1 [16]. Vaspin has been suggested to be a ‘good adipocytokine’ [16,42]. Vaspin has an anti-apoptotic effect in endothelial cells [19,26,43], and anti-inflammatory [29], and anti-migratory effects in vascular smooth muscle cells [44]. Taking the results of these previous studies and the current study together, we propose that vaspin plays a protective role in the pathogenesis of atherosclerosis.

The serum level of vaspin in human varies according to the characteristics of the studied subjects [45-50]. In view of atherosclerosis, the vaspin levels varied among studies [50-52]. It has been suggested that an elevated level of vaspin is a compensatory factor in subjects with obesity or insulin resistance [17,53,54]. Considering the beneficial effect of vaspin on vascular cells demonstrated by previous studies [19,26,29,43,44], and this study, our data provides further evidence that vaspin is a compensatory factor.

It has been demonstrated that serum levels of vaspin measured with radioimmunoassay method varied from 0.2 to nearly 2 ng/ml in subjects with normal fasting plasma glucose and from 0.3 to nearly 3 ng/ml in subjects with type 2 diabetes [53]. In our study, vaspin concentrations more than 25 ng/ml showed the anti-inflammatory (Figure 2C) and inhibitory effects against TNFα-induced monocyte adhesion to HAECs (Figure 4). These findings suggest that a higher concentration of vaspin might be needed to ameliorate the endothelial inflammatory status.

In conclusion, vaspin might attenuate cytokine-induced gene expression of adhesion molecule by inhibiting NF-κB following AMPK activation. These results provide a novel molecular mechanism underlying the anti-atherogenic effect of vaspin in endothelial cells. Further studies are needed to elucidate the specific mechanism by which vaspin activates AMPK in vascular endothelial cells.

### Significance

Although vaspin, a recently identified adipocytokine in visceral adipose tissue of OLETF rat, has been suggested to have insulin sensitizing effect, its function in the body is largely unknown, especially in vascular cells. Previously, several studies had demonstrated that vapsin exerted anti-atherogenic effect on vascular cells. The present study provides a novel molecular mechanism that vaspin inhibits cytokine-induced expression of adhesion molecule genes by inhibiting NF-κB following AMPK activation. This study is thought to be meaningful in that it showed the novel function of vaspin in vascular cells and added another evidence supporting that vaspin acted as a compensatory factor.

## Abbreviations

ACC: Acetyl-CoA carboxylase; AMPK: AMP-activated kinase; Ct: Cycle value; eNOS: Endothelial nitric oxide synthase; HAEC: Human aortic endothelial cell; ICAM-1: Intracellular adhesion molecule-1; IL: Interleukin; IκB: Inhibitor of nuclear factor-κB; MCP-1: Monocyte chemoattractant protein-1; NF-κB: Nuclear factor-κB; NO: Nitric oxide; PI3-kinase: Phosphatidylinositol3-kinase; SEM: Standard error of mean; siRNA: Small interfering RNA; TNFα: Tumor necrosis factor α; VCAM-1: Vascular adhesion molecule-1.

## Competing interests

The authors declare that they have no competing interests.

## Authors’ contributions

CHJ, HKY, YLL carried out the cell culture, performed the molecular experiments, participated in the design of the study, and drafted the manuscript. CHJ, and YLL carried out the Western blot analysis and the real time PCR. S-WK and WJL helped to draft the manuscript. MJL, and YMK carried out the reagent preparation and the statistical analysis. HKY, and YLL participated in the molecular experiments. J-YP conceived of the study, and participated in its design and coordination and helped to draft the manuscript. All authors read and approved the final manuscript.

## Supplementary Material

Additional file 1: Figure S1 GRP78 mediates the AMPK activation by vaspin. The effect of vaspin on the activation of AMPK and ACC were measured at 1 hr after treatment with vehicle or vaspin (100 ng/ml). HAECs were transfected with 10 nM control siRNA or anti-GRP78 siRNA (1071402, Bioneer, Daejeon, Korea) using LipofectAMINE2000 (Invitrogen) 48 hr before above treatment. Levels of phosphorylated AMPK and ACC were normalized against total levels of AMPK and ACC. The expression of GRP78 was determined using anti-GRP78 antibody (sc-1050, Santa Cruz Biotechnology, 1:1000). Data are shown as mean ± SEM of three independent experiments. *p<0.05 vs. untreated cells (Control), †*p*<0.05 vs. cells treated with control siRNA+100 ng/mL of vaspin. Click here for file
