# Supplementary material for: Vaspin inhibits cytokine-induced nuclear factor-kappa B activation and adhesion molecule expression via AMP-activated protein kinase activation in vascular endothelial cells
Source: Cardiovasc Diabetol. 2014 Feb 12;13:41. doi: 10.1186/1475-2840-13-41 (PMC3925442; doi:10.1186/1475-2840-13-41)
Supplement: Additional file 1: Figure S1 — GRP78 mediates the AMPK activation by vaspin. The effect of vaspin on the activation of AMPK and ACC were measured at 1 hr after treatment with vehicle or vaspin (100 ng/ml). HAECs were transfected with 10 nM control siRNA or anti-GRP78 siRNA (1071402, Bioneer, Daejeon, Korea) using LipofectAMINE2000 (Invitrogen) 48 hr before above treatment. Levels of phosphorylated AMPK and ACC were normalized against total levels of AMPK and ACC. The expression of GRP78 was determined using anti-GRP78 antibody (sc-1050, Santa Cruz Biotechnology, 1:1000). Data are shown as mean ± SEM of three independent experiments. *p<0.05 vs. untreated cells (Control), †p<0.05 vs. cells treated with control siRNA+100 ng/mL of vaspin. [file 1475-2840-13-41-S1.pdf]

Online Supplement

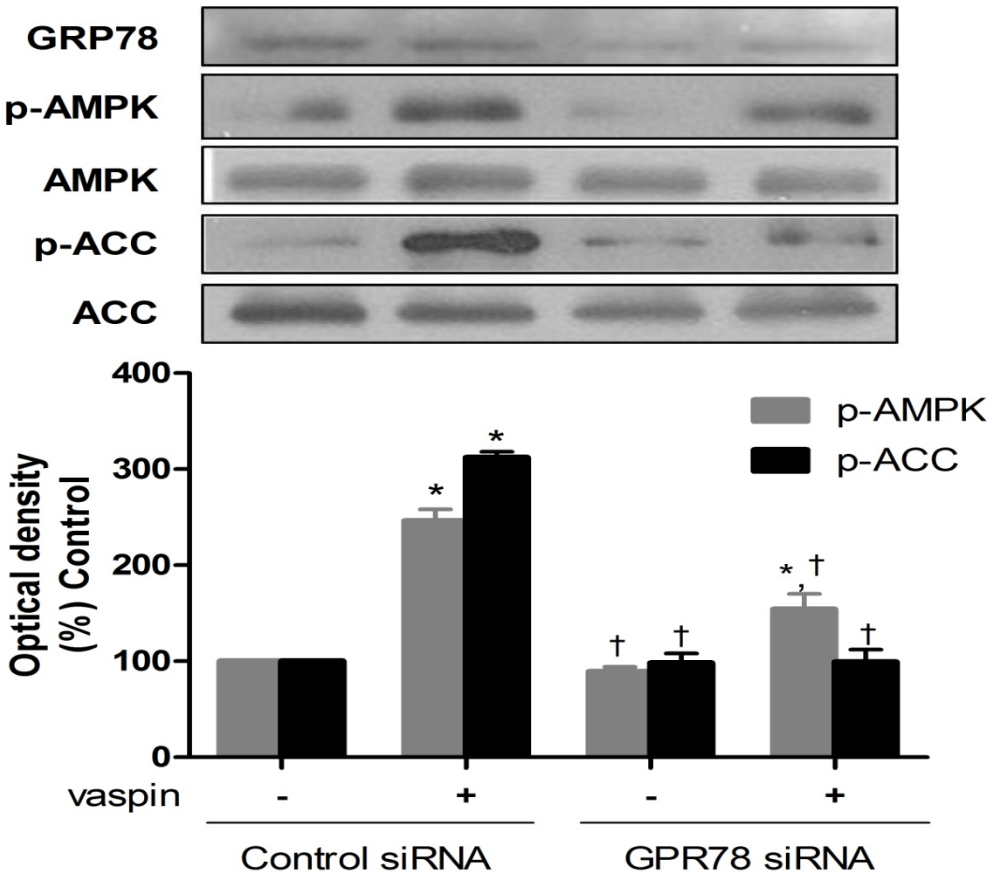

Figure I. GRP78 mediates the AMPK activation by vaspin. The effect of vaspin on the activation of AMPK and ACC were measured at 1 hr after treatment with vehicle or vaspin (100 ng/ml). HAECs were transfected with 10 nM control siRNA or anti-GRP78 siRNA (1071402, Bioneer, Daejeon, Korea) using LipofectAMINE2000 (Invitrogen) 48 hr before above treatment. Levels of phosphorylated AMPK and ACC were normalized against total levels of AMPK and ACC. The expression of GRP78 was determined using anti-GRP78 antibody (sc-1050, Santa Cruz Biotechnology, 1:1000). Data are shown as mean ± SEM of three independent experiments. $^{*}p<0.05$ vs. untreated cells (Control), $^{\dagger}p<0.05$ vs. cells treated with control siRNA+100 ng/mL of vaspin.
